# Multi-project wafers for flexible thin-film electronics by independent foundries

**DOI:** 10.1038/s41586-024-07306-2

**Published:** 2024-04-24

**Authors:** Hikmet Çeliker, Wim Dehaene, Kris Myny

**Affiliations:** 1 ESAT, KU Leuven, Leuven, Belgium; 2 imec, Leuven, Belgium

**Keywords:** Electrical and electronic engineering, Nanoscience and technology, Electronics, photonics and device physics

## Abstract

Flexible and large-area electronics rely on thin-film transistors (TFTs) to make displays^1–3^, large-area image sensors^4–6^, microprocessors^7–11^, wearable healthcare patches^12–15^, digital microfluidics^16,17^ and more. Although silicon-based complementary metal–oxide–semiconductor (CMOS) chips are manufactured using several dies on a single wafer and the multi-project wafer concept enables the aggregation of various CMOS chip designs within the same die, TFT fabrication is currently lacking a fully verified, universal design approach. This increases the cost and complexity of manufacturing TFT-based flexible electronics, slowing down their integration into more mature applications and limiting the design complexity achievable by foundries. Here we show a stable and high-yield TFT platform for the fabless manufacturing of two mainstream TFT technologies, wafer-based amorphous indium–gallium–zinc oxide and panel-based low-temperature polycrystalline silicon, two key TFT technologies applicable to flexible substrates. We have designed the iconic 6502 microprocessor in both technologies as a use case to demonstrate and expand the multi-project wafer approach. Enabling the foundry model for TFTs, as an analogy of silicon CMOS technologies, can accelerate the growth and development of applications and technologies based on these devices.

## Abstract (web summary)

The iconic 6502 microprocessor designed in two key thin-film transistor technologies by independent foundries is used to demonstrate and expand the multi-project wafer approach for flexible electronics.

## Main

The display manufacturing industry operates as an integrated device manufacturer (IDM), in which display companies are entirely vertically integrated and responsible for all aspects of the product, including design, intellectual property, technology manufacturing, business models and sales. This business model succeeds when design requests and modifications remain relatively simple and fall within the scope and expertise of current design and application teams. The current mode of operation jeopardizes the numerous application possibilities for thin-film electronics. Different applications require distinctive design strategies and know-how, leading to the need for an increased number of designers in these technologies. Moreover, the thin-film transistor (TFT) technology affordability of the fabless design groups and companies will be very crucial in the success towards the increasing application window. Therefore, to decrease the cost, the dies in the wafer can be divided into smaller sections or sub-dies and sold independently as separate projects, making the multi-project wafer concept for TFTs the essential and next big step to grow the field.

The interface between a foundry and a design house is a process design kit (PDK). This PDK provides the technical details of the foundry technology node, including a simulation model and all setup and verification files supporting varying design tools. Although PDKs for TFTs may still be at their infancy, especially towards the creation of a stable and accurate simulation model, this is expected to grow in future as the number of users increases.

The revolutionary introduction of the foundry-mode access combined with the multi-project wafer concept would be a true game changer for the flexible electronics industry. It will pave the way to increase the design complexity for TFT technologies and open the path towards application research, enabling the Internet-of-Things (IoT) era, the Internet-of-Everything and wearable healthcare, requiring mass production of a very large number of TFT-based electronic circuits.

## Historical perspective

The prospects can be found in the analogy of the silicon complementary metal–oxide–semiconductor (CMOS) chip industry, which initially operated in an IDM mode. One of the main breakthroughs in the late 1980s came when the Taiwan Semiconductor Manufacturing Company (TSMC) established the foundations of the fabless business model^18,19^. As such, foundries can focus on the manufacturing of the CMOS chips and the development of the next-generation technology nodes, whereas external, fabless, design houses can focus on the design activities from a research and business perspective.

Typical TFT foundries emerge from the display manufacturing business, using a large glass plate—covered with a flexible polyimide film—as a substrate that has evolved from several different generations ranging between GEN1 (300 mm × 400 mm) and GEN10.5 (2.94 m × 3.37 m). The traditional photolithography equipment for those foundries exhibits critical dimensions in the micrometre range, yielding transistors with above-µm channel lengths. Developing technology demonstrators and proof of concepts on a small scale requires the fabrication of only a limited amount of panels, in which the cost is dictated by the non-recurring engineering cost, comprising amongst others the photolithography masks, advocating for the multi-project wafer approach.

Another type of TFT foundries inspired by the Si CMOS industry uses a wafer-based substrate concept with similar wafer sizes ranging from 150 mm to 300 mm diagonal. To make this technology cost-effective, TFT technologies developed on those substrates are modestly scaled towards a few hundreds of nanometres, avoiding the use of expensive photolithography equipment. Owing to the low-process temperatures, a thin flexible substrate on top of a glass carrier can be used for manufacturing. These foundries focus today on microdisplays or IoT electronics. The main advantage of these technologies is the scalability of the technology because of the available lithography toolsets, as it will enable high-density flexible circuits. The channel lengths are typically smaller for those foundries—sub-micrometre ranges—compared with the panel-based approach, with reduced overlay leading to faster operating transistors with smaller parasitics. There is a limitation in scaling depending on the semiconductor technology, whether it is available in an amorphous or polycrystalline state. Amorphous semiconductors, such as amorphous indium–gallium–zinc oxide (IGZO), have the potential for (deep) sub-micrometre channel lengths and are already being developed for back-end-of-line applications on top of Si CMOS^20^.

## TFT technologies

There are several commercially available TFT technologies, categorized by the underlying semiconductor material^21^. The first developed technology was amorphous silicon or a-Si. It became attractive as it could be manufactured on glass. a-Si is a backplane technology, used in applications in which excellent transistor performance is not essential, such as large-area imaging applications or budget-friendly LCD panels. It exhibits a charge carrier mobility of 0.5–1 cm^2^ V^−1^ s^−1^. A more recent semiconductor, invented in 2004 and enabling room temperature processing, is based on metal-oxide complexes, such as the combination of indium–gallium–zinc oxide (IGZO) (ref. ^22^). Mostly, this semiconductor comes in an amorphous state, enabling a high downscaling potential. IGZO outperforms a-Si with a charge carrier mobility of more than 10 cm^2^ V^−1^s^−1^. The main challenge of this technology is the stability under stress. Both IGZO and a-Si can be manufactured on the largest glass substrates, making them suitable for large-area applications such as large televisions and imagers. Another challenge is that both a-Si and IGZO typically yield only n-type semiconductors, limiting the design of pixels and circuits to a unipolar configuration. The absence of a complementary semiconductor imposes greater complexity and limitations in achieving low-power, high-performant circuits. However, the extremely low-leakage current of IGZO transistors^20,23^ creates innovative opportunities in circuits, especially for memory.

Low-temperature polycrystalline silicon (LTPS) is a semiconductor exhibiting typical charge carrier mobilities of more than 50 cm^2^ V^−1^s^−1^ for the p-type and more than 100 cm^2^ V^−1^s^−1^ for the n-type devices, making it the best performant TFT-based semiconductor on glass and flex^24^. Some application fields today are high-resolution displays for smartphones and high-performance microdisplays. The polycrystalline nature of the semiconductor makes it less interesting for sub-micrometre downscaling because of decreased performance and variability.

## Multi-project wafer concept with flexible 6502 use case

**Fig. 1:**
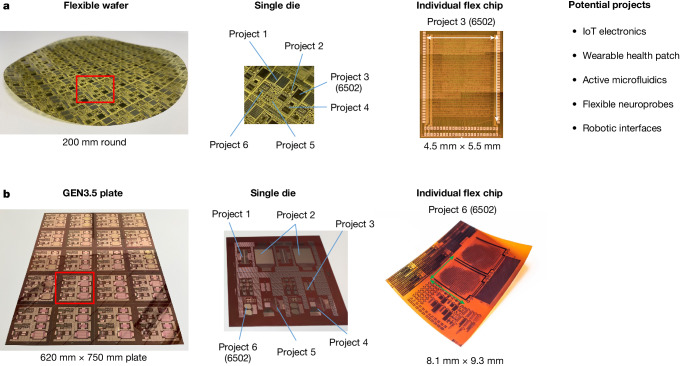
The multi-project wafer approach for TFTs. **a**, Round wafers (200  mm) offering high-density unipolar IGZO transistors. **b**, GEN3.5 plates offering complementary LTPS transistors. The substrate has been divided into smaller dies with individual projects. The focus of this work is on the 6502 microprocessor that has been taped out in both technologies. Panel **a**, image ‘Project 3 (6502)’, adapted with permission from ref. ^10^, IEEE.

This work demonstrates the foundry model for flexible TFT technologies by designing the iconic 6502 microprocessor directly in two independent foundries—wafer-based and plate-based. The first TFT technology is based on a 200-mm round wafer approach of Pragmatic offering 0.8 µm IGZO transistors with extra metallization layers to enable higher density routing. The 6502 chip was a sub-project within the single die, as indicated in Fig. 1a. The second TFT technology is a plate-based option offering 3 µm complementary LTPS transistors. The technology of PanelSemi offers a small die of GEN-3.5 TFT panels (size 620 × 750 mm^2^) as base modules for the multi-project approach. Also here, Fig. 1b shows the individual 6″ die that is repeated several times across the plate, in which the 6502 is present as a sub-project. Although our recent IGZO 6502 chip has already been partially published at ISSCC22 (ref. ^10^), it is included in this work as a direct comparison with the 6502 plate-based chip to provide a more complete and comprehensive evaluation of foundry possibilities for flexible TFT electronics. More TFT-based companies are currently considering offering multi-project plates, such as LinkZill or Tianma, but those have not been studied in this work. Furthermore, we anticipate that more display companies and TFT foundries may adopt the presented and studied model, following the historical precedent of Si CMOS.

The 6502 processor was one of the first commercial microprocessors introduced in 1975 and integrated into many products of the 1980s, such as the Nintendo Entertainment Systems, the Commodore 64 and the Apple II (ref. ^25^). The original version, fabricated in an 8-µm nMOS-only technology, consists of 3,510 enhancement-mode transistors and 1,018 depletion-load pull-up transistors, adding up to 4,528 devices. The microprocessor had an 8-bit data bus and 16-bit address bus, operating at 1 MHz. Owing to the legendary nature of this processor and all its ground-breaking aspects in application and microprocessor research from that time onwards, we have selected this chip to use as a vehicle to demonstrate the foundry mode with TFTs.

The logic topology for digital gates must be optimally designed according to the foundry specifications, either offering unipolar or complementary transistor technologies. These logic topologies are depicted in Supplementary Fig. 1. The most straightforward design option is a complementary inverter, which can be realized by the combination of LTPS nMOS and pMOS transistors. As classically known, this is the most robust solution, yielding the least power consumption. As TFT technologies do not always offer complementary semiconductors, many studies have been performed on inverter implementations for unipolar IGZO transistors. On the basis of these, we have opted for a pseudo-CMOS approach^26,27^. This requires four transistors and one extra power rail to realize an inverter. This is different from the original MOS devices in the 1970s, in which local doping profiles can yield enhancement and depletion devices. The used circuit topology at that time is also depicted in Supplementary Fig. 1, which is an interesting logic style imposing fewer transistors for complex gates, even compared with CMOS inverters, at the cost of static power consumption. A two-input NOR gate uses three transistors for the depletion-load nMOS logic style, six n-type IGZO transistors for the pseudo-CMOS implementation and four LTPS complementary transistors. IGZO or unipolar TFT technologies could also benefit from the availability of a second threshold voltage, created by technology options, such as local doping by additional treatments^28–30^ or by the introduction of a second gate^26^, which, after maturation can be offered through the foundry access.

Circuit schematics of the IGZO pseudo-CMOS and LTPS CMOS inverters are shown in Fig. 3, including the selected device sizes in µm. For pseudo-CMOS logic, the input port of the inverter is connected to the gate terminals in the pull-down network to form the logic function, whereas other TFTs play the part of the pull-up network (Fig. 3). For complementary logic with LTPS technology, n-type and p-type devices with 3 µm channel length are used. The channel width ratio is selected to be 2:1 (p-TFT:n-TFT) for obtaining a symmetrical drive strength of pull-down and pull-up similar to that for Si CMOS inverters.

**Fig. 2:**
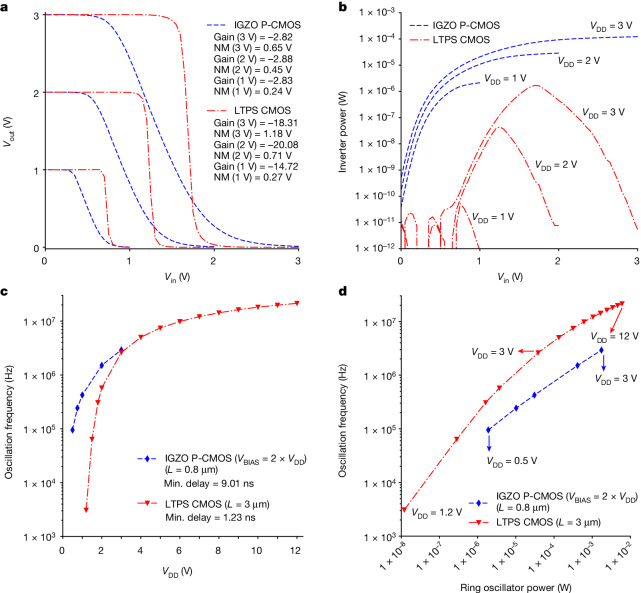
Inverter and ring oscillator characterization. **a**, Measured voltage transfer characteristics of IGZO pseudo-CMOS and LTPS CMOS inverters for various *V*_DD_ supplies. **b**, Measured power in logarithmic scale compared with input voltage of inverters. **c**, Measured ring oscillator frequency compared with its supply voltage for both technologies. **d**. Corresponding frequency versus power plot for each technology. P-CMOS, pseudo-CMOS; Min., minimum; NM, noise margin.

Figure 2a shows the measured d.c. characteristics of both inverter architectures for various supply voltages (*V*_DD_), ranging from 1 V to 3 V. Correct operation of a pseudo-CMOS inverter relies on the condition that the second supply voltage (*V*_BIAS_) is equal to or at least one *V*_T_ larger than *V*_DD_, yielding a rail-to-rail swing of the output voltage. Moreover, increasing the *V*_BIAS_ shifts the voltage transfer curve in the right direction, which can be useful for post-fabrication tuning of the static noise margin, depending on the local *V*_T_ and variability values^10^. Figure 2a shows the voltage transfer curves in which *V*_BIAS_ equals twice *V*_DD_ for pseudo-CMOS logic. Figure 2b shows the logarithmic power consumption curves with respect to *V*_in_ for all logic styles, in which a difference in static power consumption of more than six orders of magnitude is observed. The static power when *V*_in_ is logic 0 stems from the leakage of n-type devices because of the *V*_T_ difference in technologies.

To compare the performance and power consumption metrics of both inverter architectures, 19-stage ring oscillator circuits are designed and characterized (Supplementary Fig. 3). Figure 2c shows the oscillation frequency values with respect to *V*_DD_ supply, in which V_BIAS_ equals twice *V*_DD_ for pseudo-CMOS. The CMOS inverter can achieve a stage delay lower than 1.5 ns when operated at large supply voltages. The oscillation frequency of those ring oscillator inverters is plotted versus the power consumption in Fig. 2d. The LTPS CMOS inverter consumes less power regardless of supply voltage because of its complementary structure.

**Fig. 3:**
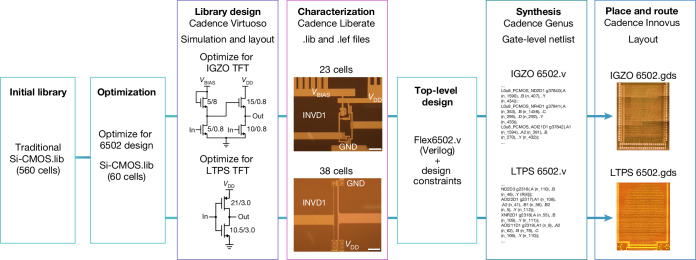
Representation of the digital flow implemented for IGZO and LTPS technologies. Lots of similarities can be observed. The main difference is the complexity of the cell library, being more complex for LTPS because of the benefits of complementary logic gates. Scale bars, 20 μm. Image ‘IGZO 6502.gds’ adapted with permission from ref. ^10^, IEEE.

**Fig. 4:**
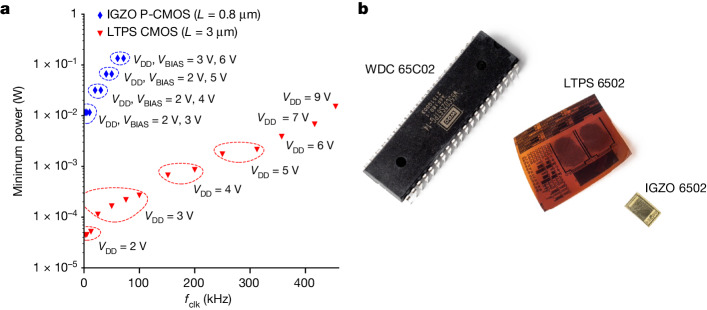
Flex 6502 chips and characterization. **a**, The minimum power consumption of the characterized 6502 chips to reach different clock frequencies (*f*_clk_). **b**, Photograph of all three chips at once: the vintage WDC 65C02 in a 40-pin DIP package (left), the flex LTPS 6502 (middle) and the flex IGZO 6502 (right)^10^.

We have subsequently designed and realized 6502 chips in both available foundry technologies, according to the industry-ready design flow, starting from a traditional CMOS library with a strong focus on optimizing cells frequently used for the 6502 design. This is schematically represented in Fig. 3, and more details on the flow are explained in the Methods. Both chips have been characterized with a 48-pin probe card after the fabrication cycle, without dicing and delamination. The probe card includes level shifters to enable verification with an FPGA that generates the clock, the 64 kB memory and universal asynchronous receiver–transmitter (UART) (for more details, see the Methods). Figure 4a shows the resulting minimum power consumption that is required to reach different clock frequencies, up to 454 kHz. It is unfair and not necessary to directly compare both chips because of the absence of a complementary semiconductor in IGZO and more importantly because of the different application cases for both technologies. Nonetheless, both of them are fully functional and show predictable specifications related to their underlying transistor technology.

Table 1 summarizes the results of the 6502 microprocessor implementations for all discussed technologies—namely, the original nMOS, the n-type IGZO (ref. ^10^) and the newest CMOS LTPS version until now. Impressively, the original MOS6502 yields the lowest transistor count, which may differ because of an optimized version in the 1970s compared with an open-source version used for both the flex implementations. Moreover, in those early days, the only possibility to realize a design was a full-custom flow, in which the semi-custom flow nowadays is mandatory to maintain a reasonable design time while creating some overhead. Another difference is the number of transistors per logic gate, which is higher for both flex implementations. Moreover, the arithmetic and logic unit has not been optimized for the flexible 6502 versions, and traditional standard cells have been used. Table 1 also reports the characterized yield of functional 6502 implementations in both technologies. We observed a 42% yield of the Flex IGZO 6502 in Pragmatic technology with an unoptimized design for yield. We did not focus on any design for manufacturing approach to increase the yield figure as it was out of scope for our research. The yield has been determined by characterizing 104 chips across three wafers. A more thorough analysis of yield figures in Pragmatic technology as a function of chip complexity has been studied recently^11^. Similarly, for LTPS, we have measured 100 samples over several GEN3.5 plates and achieved a yield of 89%. A typical coloured die map can be seen in Supplementary Fig. 5. Figure 4b shows a photo of all microprocessors at once. Both chips show the key advantages of the multi-project wafer concept for both wafer-based and plate-based technologies. Supplementary Fig. 6 details the yield data specifically per frequency for 85 evaluated LTPS 6502 processors. Each processor worked at 357.1 kHz, whereas only a selection can be operated at 416.7 kHz. The lowest fraction, 23 processors, showed correct behaviour at 454.5 kHz.

**Table 1:** Comparison of both flex 6502 implementations with the original design

	MOS 6502	Flex IGZO 6502 (ref. ^10^)	Flex LTPS 6502
Substrate size	3 inch (76.2 mm round)	200 mm round	620 mm × 750 mm (GEN-3.5)
Substrate material	Silicon	Glass + polyimide	Glass + polyimide
Semiconductor	Si nMOS	IGZO	LTPS
Transistors	nMOS only	n-type only	nMOS + pMOS
Minimum channel length	8 µm	0.8 µm	3 µm
Chip size	5.4 mm × 5.4 mm	4.5 mm × 5.5 mm	8.1 mm × 9.3 mm
Number of transistors	4,528	16,392	12,628
Density (transistors per mm^2^)	155 devices	662 devices	161 devices
Clock frequency	1 MHz	71.4 kHz	454.5 kHz
Power consumption at that frequency	250 mW (5 V)	134.9 mW (6 V)	15.3 mW (9 V)
Minimum power consumption	Not available	11.6 mW (3 V)	44.6 µW (2 V)
Yield	Not available	42%	89%
Year of introduction	1975	2022	2024

The integration density is the highest for the flex IGZO implementations, which can be attributed to the best photolithography resolution and the available extra metallization layers used enabling over-the-cell routing. This TFT technology will, therefore, enable the highest density circuits for flexible IoT applications or for hybrid circuits combining Si CMOS and high-density IGZO electronics. Owing to the amorphous nature of the semiconductor and the wafer-based approach with conventional semiconductor lithography equipment, IGZO transistors have the potential to follow a modest scaling approach for flexible electronics. The term ‘modest’ has been carefully selected to keep the manufacturing process flow as simple as possible to maintain the main benefits of this technology. IGZO scaling would enable better performing, lower power and even higher density circuits, whereby, in addition, a second threshold voltage or a p-type counterpart would create a substantial impact. The application field for wafer-based TFT electronics should be found in the high-density electronics domain on glass or flexible substrates, such as IoT chips, wearable patches, high-density arrays for microLED or microfluidics and many more.

The flex LTPS implementation shows great benefits in terms of clock frequencies and respective power consumption, stemming from a larger charge carrier mobility and complementary technology. The newest flexible LTPS chip exhibits a maximum clock frequency of 454.5 kHz, which is only twice less than the original commercial chip, making it already a viable chip for various applications. The display plate-based platform based on LTPS cannot be on par with IGZO with respect to scaling because of the polycrystalline nature of the semiconductor, although a previous study has discussed 200 nm channel lengths for LTPS^31^. Moreover, the photolithography equipment to fabricate display plates has limitations in the µm range to keep it cost-effective. The application field for plate-based circuits should be found in large-area electronics applications, which include large arrays of sensors or actuators, microfluidics-based lab-on-a-chip or cm-sized wearable patches, which would require large-area electronics.

## Conclusion

We have demonstrated the multi-project wafer concept for flexible TFT technologies, enabling fabless design companies and research institutions to start experimenting with those emerging technologies for a large variety of applications. The multi-project wafer concept has been instrumental for the growth of the Si CMOS chip industry since the late 1980s till today, in which Si chips play an important part in our daily lives. Similarly, TFTs that are mainly commercially available today in many displays and imagers may find various applications, such as the IoT, wearable healthcare, e-skin, smart robotics, lab-on-a-chip and many more. To demonstrate this concept, the iconic 6502 microprocessor has been designed in two different flexible thin-film transistor technologies—namely, on a 200-mm wafer-based, 0.8 µm IGZO technology node and a GEN-3.5 620 mm ×750 mm plate-based 3 µm LTPS technology.

The flexible IGZO processor has the best integration density, to our knowledge, because of the transistor technology resolution and the extra metallization layers for routing, making the wafer-based approach suitable for applications requiring low-area high-density TFT circuits. The LTPS version, with complementary n-type and p-type transistors, yields clock frequencies almost on par with the original MOS6502, serving many applications in the late 1980s.

These results demonstrate the viability of transistor technologies on flexible substrates for serving a broad range of applications beyond their traditional display and imager consumer products. The introduction of the multi-project wafer concept for flexible thin-film transistor technologies combined with the demonstrated specs of both 6502 chips is expected to pave the way towards an era of performing research and development in those technologies leading to previously unknown and unprecedented applications.

## Methods

### Manufacturing

The main purpose of this work is to demonstrate the foundry-based model. Therefore, the manufacturing of both 6502 chips has been outsourced to foundries, Pragmatic and PanelSemi. Pragmatic has a 200-mm wafer approach, in which a thin flexible polyimide layer is applied to the glass carrier during processing. The LTPS chip has been outsourced by the foundry interface PanelSemi and was manufactured on a GEN3.5 plate. More details about both options are detailed in Supplementary Table 1. The IGZO technology has two extra routing layers available enabling over-the-cell routing, whereas LTPS has a limitation of one extra metal. More process information on the IGZO transistor is provided in a previous work^9^. The transistor transfer characteristics for both technologies are shown in Supplementary Fig. 2.

### Process design kit

The PDK is the interface between the design and the manufacturing. The PDK consists of various files to validate the design, such as layout versus schematic (LVS) or design rule checks (DRC). Moreover, models describing the devices are used during the design and simulation phase. PDKs for TFT technologies are still at their infancy; nonetheless, Pragmatic has offered a PDK with a device model, DRC and LVS. As this model is new to PanelSemi and the display field, we have been realizing a first-generation PDK for the LTPS technology. First, we created a simple Verilog-A model from the transistor measurements shown in Supplementary Fig. 2 to enable circuit simulations. Several TFT-based models for varying TFT semiconductors have been discussed in the literature^32–36^. Second, based on the design rules provided by PanelSemi, we have created a basic DRC and LVS deck to validate our layouts.

### Semi-custom design flow

We have set up a semi-custom digital design flow for IGZO and LTPS technologies. A simplified diagram of the design flows that have been implemented is shown in Fig. 3. A Si CMOS standard cell library consisting of 560 cells (CMOS.lib) has been selected as starting point to redesign and optimize a selection of these cells for IGZO and LTPS technologies. Our final library files comprise 23 cells for IGZO PCMOS.lib and 38 cells for LTPS CMOS.lib after eliminating the cells that are not crucial or not used in the final design. In future, this library can be further optimized with an increased number of cells. These .lib files contain detailed information about every standard cell in terms of power consumption, speed performance and area. Flex6502.v is the top-level Verilog file that is based on an open-source version of MOS 6502 microprocessor^37^. Finally, the top-level layout of the microprocessors ready for tape out is available after synthesis and standard cell place and route.

### Measurement setup

For verification of flexible 6502 microprocessors, we have designed a measurement setup that consists of three main parts: a 48-pin probe card, voltage level shifters and an Artix-7 FPGA board with UART connection. The pins of the probe card land on the pads of the design under test (DUT), which is kept on the wafer without delaminating the flexible substrate from the glass. The power supplies of the DUT (*V*_DD_ and *V*_BIAS_), the level shifters and the FPGA are all connected to separate source measurement units through the main board so that the *I*_DD_ and *I*_BIAS_ values are measured in real time and saved to the control personal computer by a GPIB cable.

Each input and output signal of the 6502 chip is accompanied by an inverting level shifter. This is essentially a resistor-load MOSFET (metal-oxide-semiconductor field-effect transistor) inverter with operational amplifier buffers, situated on both the DUT and FPGA sides. The supply voltage of the level shifters is equal to the *V*_DD_ of the DUT but connected with a separate power supply to enable accurate extraction of the power consumption of the individual 6502 chip. As the level shifters invert the digital signal, another inversion of inputs and outputs is done on the FPGA side.

The external components of the 6502 microprocessor are provided inside the FPGA, including a 64-kB memory, a clock signal generator and an UART communication interface with the computer. This enables control over the operating speed of the chip and facilitates real-time read and write operations for the data. We developed a test program in assembly language, using key instructions such as ‘arithmetic operations’, ‘load’, ‘store’, ‘branch’ and many more. This assembly is subsequently compiled into opcodes and uploaded into the block RAM of the FPGA. To verify whether the test program is executed correctly, we used an available pin of the FPGA (compare bit) such that it is toggled to logic-1 when the address and data busses of the 6502 have the pre-coded values that can be achieved only if the program runs successfully. Supplementary Fig. 4. demonstrates the waveforms captured by the oscilloscope for the program, which calculates the square root of 9. Later, to prove complete functionality, we ran the assembly code of the popular Snake game on the microprocessor and played it in real-time^10^.

## Online content

Any methods, additional references, Nature Portfolio reporting summaries, source data, extended data, supplementary information, acknowledgements, peer review information; details of author contributions and competing interests; and statements of data and code availability are available at 10.1038/s41586-024-07306-2.

### Supplementary information

Supplementary Information Supplementary Figs. 1–6 and Supplementary Table 1.

## Data Availability

All data presented in the paper are available from the corresponding author upon request.

## Code availability

The code of the 6502 programs to validate the chips is available from the corresponding author upon request.

## References

[1] Sanford JL, Libsch FR (2003). 4.2: TFT AMOLED pixel circuits and driving methods. SID Symp. Dig. Tech. Pap..

[2] Nathan A, Chaji GR, Ashtiani SJ (2005). Driving schemes for a-Si and LTPS AMOLED displays. J. Disp. Technol..

[3] Iwase Y (2018). A novel low-power gate driver architecture for large 8 K 120 Hz liquid crystal display employing IGZO technology. J. Soc. Inf. Disp..

[4] Lujan RA, Street RA (2012). Flexible X-ray detector array fabricated with oxide thin-film transistors. IEEE Electron Device Lett..

[5] Street, R. A., Lu, J. P., Bert, J., Strnad, M. & Antonuk, L. E. TFT backplane technologies for advanced array applications. In *2015 IEEE International Electron Devices Meeting (IEDM)* 6.4.1–6.4.4 (IEEE, 2015).

[6] van Breemen AJJM (2020). Curved digital X-ray detectors. npj Flex. Electron..

[7] Kurokawa Y (2008). UHF RFCPUs on flexible and glass substrates for secure RFID systems. IEEE J. Solid-State Circuits.

[8] Myny K (2014). A thin-film microprocessor with inkjet print-programmable memory. Sci Rep..

[9] Biggs J (2021). A natively flexible 32-bit Arm microprocessor. Nature.

[10] Çeliker, H., Sou, A., Cobb, B., Dehaene, W. & Myny, K. Flex6502: a flexible 8b microprocessor in 0.8 µm metal-oxide thin-film transistor technology implemented with a complete digital design flow running complex assembly code. In *2022 IEEE International Solid-State Circuits Conf.* 272–274 (ISSCC, 2022).

[11] Bleier, N. et al. FlexiCores: low footprint, high yield, field reprogrammable flexible microprocessors. In *Proc. 49th Annual International Symposium on Computer Architecture* 831–846 (Association for Computing Machinery, 2022).

[12] Takei K, Honda W, Harada S, Arie T, Akita S (2015). Toward flexible and wearable human-interactive health-monitoring devices. Adv. Healthc. Mater..

[13] Khan Y, Ostfeld AE, Lochner CM, Pierre A, Arias AC (2016). Monitoring of vital signs with flexible and wearable medical devices. Adv. Mater..

[14] Zulqarnain M (2020). A flexible ECG patch compatible with NFC RF communication. npj Flex. Electron..

[15] Lin M, Hu H, Zhou S, Xu S (2022). Soft wearable devices for deep-tissue sensing. Nat. Rev. Mater..

[16] Hadwen B (2012). Programmable large area digital microfluidic array with integrated droplet sensing for bioassays. Lab Chip.

[17] Wang D (2022). Thin-film transistor arrays for biological sensing systems. Flex. Print. Electron..

[18] Chang, M. Foundry future: challenges in the 21st century. In *2007 IEEE International Solid-State Circuits Conf. Digest of Technical Papers* 18–23 10.1109/ISSCC.2007.373573 (IEEE, 2007).

[19] Chang M (2011). Pure play. IEEE Solid-State Circuits Mag..

[20] Kunitake, H. et al. High thermal tolerance of 25-nm c-axis aligned crystalline In-Ga-Zn oxide FET. In *2018 IEEE International Electron Devices Meeting (IEDM)* 13.6.1–13.6.4 (IEEE, 2018).

[21] Ji D (2021). Recent progress in the development of backplane thin film transistors for information displays. J. Inf. Disp..

[22] Nomura K (2004). Room-temperature fabrication of transparent flexible thin-film transistors using amorphous oxide semiconductors. Nature.

[23] Sekine Y (2011). Success in measurement the lowest off-state current of transistor in the world. ECS Trans..

[24] Fortunato G, Pecora A, Maiolo L (2012). Polysilicon thin-film transistors on polymer substrates. Mater. Sci. Semicond. Process..

[25] Betker MR, Fernando JS, Whalen SP (1997). The history of the microprocessor. Bell Labs Tech. J..

[26] Myny K (2018). The development of flexible integrated circuits based on thin-film transistors. Nat. Electron..

[27] Huang T-C (2011). Pseudo-CMOS: a design style for low-cost and robust flexible electronics. IEEE Trans. Electron Devices.

[28] Wang M (2016). Threshold voltage tuning in a-IGZO TFTs with ultrathin SnO_*x*_ capping layer and application to depletion-load inverter. IEEE Electron Device Lett..

[29] Peng C (2022). A simple doping process achieved by modifying the passivation layer for self-aligned top-gate In-Ga-Zn-O thin-film transistors at 200 °C. Nanomaterials.

[30] Yu Z (2023). A high voltage gain inverter integrated with enhancement- and depletion-mode a-InGaZnO thin-film transistors. IEEE Trans. Electron Devices.

[31] Kim, S. Y., Baytok, S. & Roy, K. Scaled LTPS TFTs for low-cost low-power applications. In *2011 12th International Symposium on Quality Electronic Design* 1–6 (IEEE, 2011).

[32] Marinov O, Deen MJ, Zschieschang U, Klauk H (2009). Organic thin-film transistors: part I—compact DC modeling. IEEE Trans. Electron Devices.

[33] Torricelli F (2011). Transport physics and device modeling of zinc oxide thin-film transistors part I: long-channel devices. IEEE Trans. Electron Devices.

[34] Shur MS, Slade HC, Jacunski MD, Owusu AA, Ytterdal T (1997). SPICE models for amorphous silicon and polysilicon thin film transistors. J. Electrochem. Soc..

[35] Pappas, I. et al. A simple polysilicon thin-film transistor SPICE model. In *2006 25th International Conference on Microelectronics* 480–483 (IEEE, 2006).

[36] Kim DH (2012). Physical parameter-based SPICE models for InGaZnO thin-film transistors applicable to process optimization and robust circuit design. IEEE Electron Device Lett..

[37] Odintsov, O. ag_6502 processor. OpenCores https://opencores.org/projects/ag_6502 (2012).

