# Supplementary material for: Multi-project wafers for flexible thin-film electronics by independent foundries
Source: Nature. 2024 Apr 24;629(8011):335–40. doi: 10.1038/s41586-024-07306-2 (PMC11078730; doi:10.1038/s41586-024-07306-2)
Supplement: Supplementary file 1 — Supplementary Figs. 1–6 and Supplementary Table 1. [file 41586_2024_7306_MOESM1_ESM.pdf]

nature portfolio

https://doi.org/10.1038/s41586-024-07306-2

Supplementary information

Multi-project wafers for flexible thin-film electronics by independent foundries

In the format provided by the
authors and unedited

Supplementary Figures

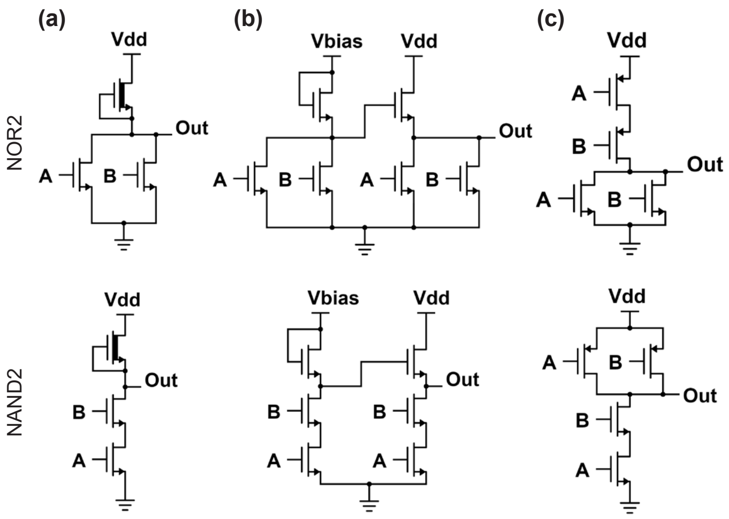

Supplementary Fig. 1

Logic topologies of NOR and NAND-gates for **a.** the original MOS6502 based on depletion and enhancement-mode devices, **b.** the flex IGZO 6502 based on unipolar single V_T n-type transistors and **c.** the flex LTPS 6502 based on complementary inverters

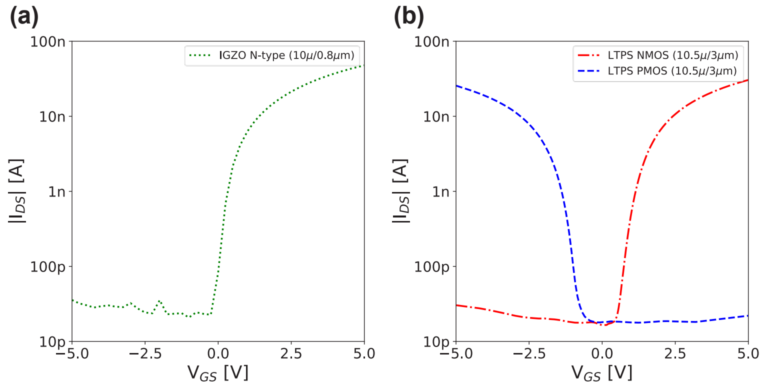

Supplementary Fig. 2

The I_D-V_{GS} transfer characteristics of a typical transistor of both foundry technologies, driven in saturation mode whereby V_{DS} equals to maximum V_{GS}. **a.** An IGZO n-type transistor with a 10μm channel width and 0.8μm channel length. **b.** nMOS and pMOS LTPS transistors plotted in the same graph, where both transistors have a channel length of 3μm and a channel width of 10.5μm. The leakage currents are limited by the measurement setup

has not been optimized for yield and can thus be improved in future runs, beyond the scope of this work.

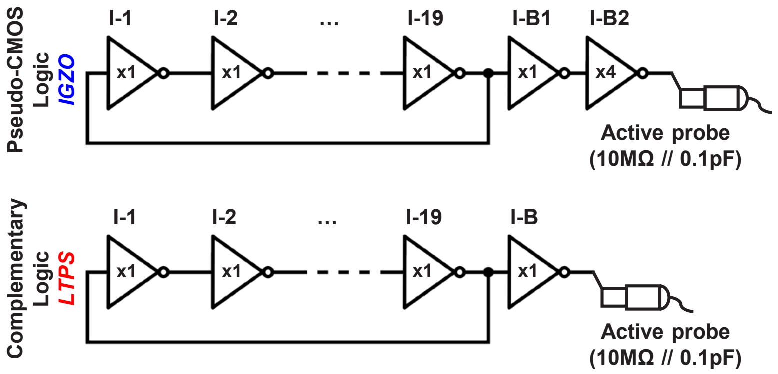

Supplementary Fig. 3

Circuit schematics of both designed ring oscillators. The IGZO ring oscillator has two inverters with size of x1 and x4 as buffer for probing, while the LTPS one has only a x1 sized inverter as buffer available. Both circuits have been measured using an active probe with impedance of 10MΩ // 0.1pF

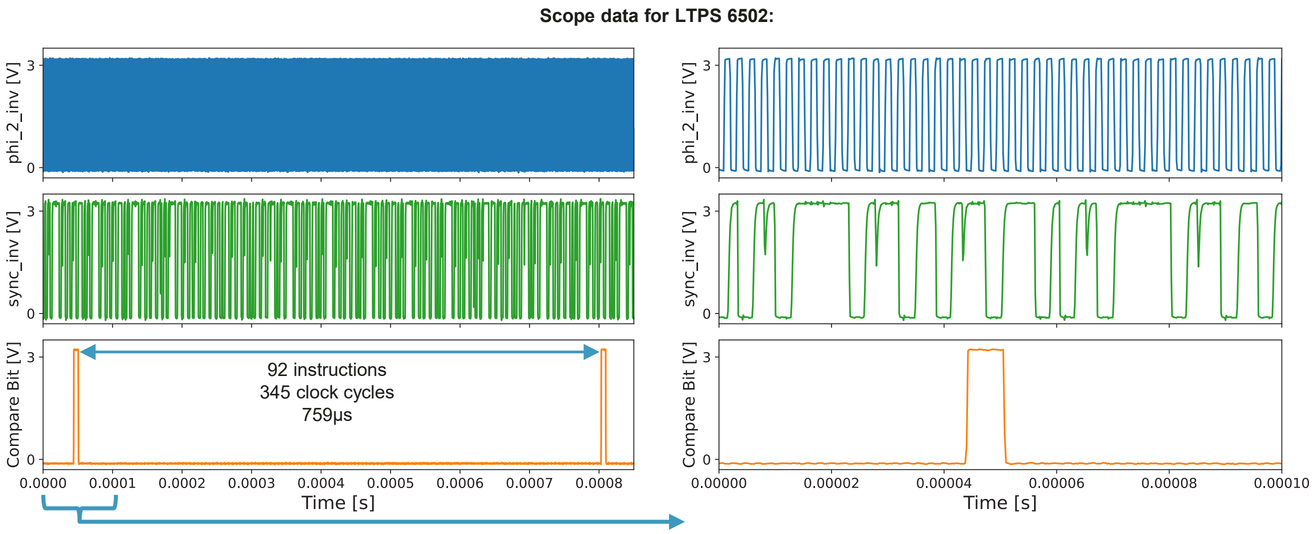

Supplementary Fig. 4

Scope data capturing 92 instructions including the compare bit for the LTPS 6502 chip. The data of the flex microprocessors is compared to the data the FPGA generates, whereafter the compare bit turns high in case all instructions are executed correctly. The right graphs are equal to the left, but display a smaller time range as indicated

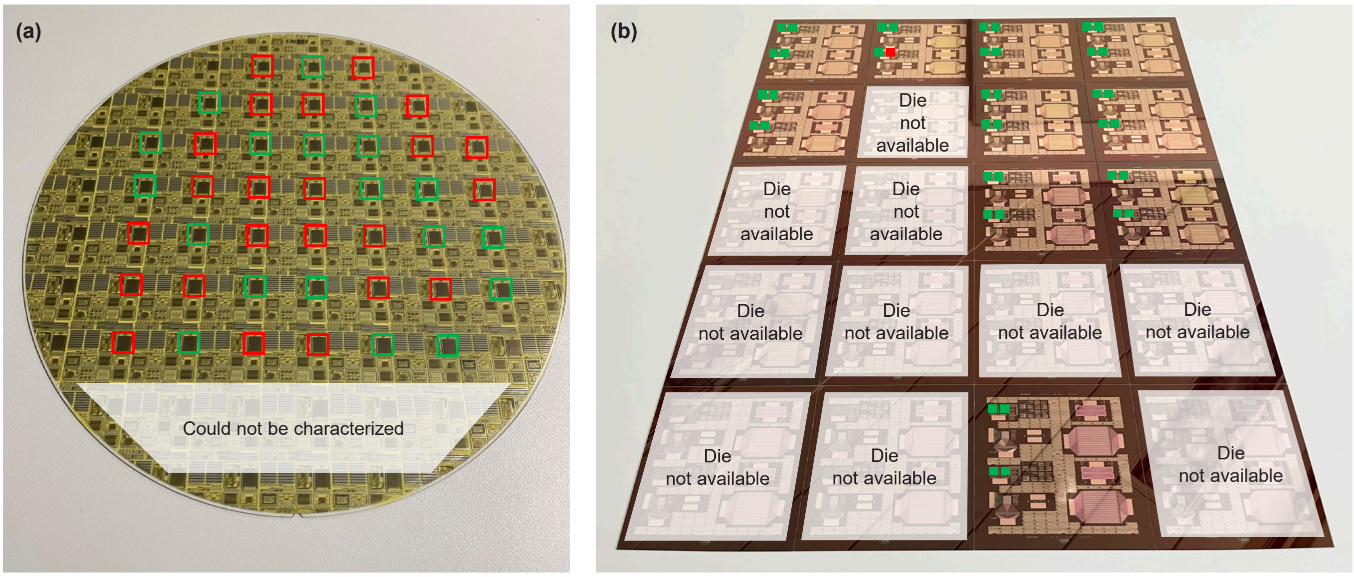

Supplementary Fig. 5

Coloured flex 6502 processor yield map characterized on **a.** a 200mm IGZO wafer and **b.** a reconstructed GEN3.5 LTPS plate from individual dies. Green implies a correct functioning processor, while red shows non-functioning 6502 chips. We have in total characterized 104 IGZO 6502 chips on 3 wafers, while 100 LTPS 6502 chips have been measured on several different plates. The chip design has not been optimized for yield and can thus be improved in future runs, beyond the scope of this work.

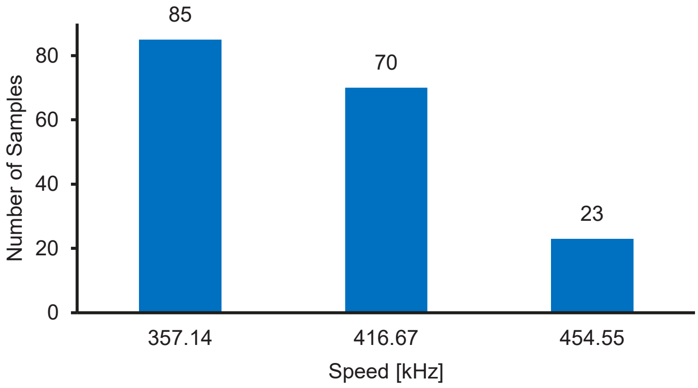

Supplementary Fig. 6

Yield data for 85 evaluated LTPS 6502 processors at different frequencies. Every chip was fully functional at 357.1kHz, while only a selection of those chips also operate at 416.7kHz or even 454.5kHz

Supplementary Tables

Supplementary Table 1. Specifications and details of both selected foundry technologies.

	**Wafer-based**	**Plate-based**
Substrate size	200mm round	620mm x 750mm (GEN-3.5)
Substrate material	Glass + PI	Glass + PI
Semiconductor	IGZO	LTPS
Transistors	n-type only	nMOS + pMOS
Number of gates	Frontgate only	Backgate and frontgate
Min channel length	0.8µm	3µm
Min transistor size	5µm / 0.8µm	10.5µm / 3µm
Number of additional metal layers beyond gate/source-drain	2	1
Footprint of an inverter [μm^2]	4080.0	6397.5
Foundry interface/contact	Pragmatic (UK)	PanelSemi (TW)
